# A Child Plexiform Neurofibroma of the Temple Region: A Case Report

**DOI:** 10.7759/cureus.60798

**Published:** 2024-05-21

**Authors:** Mubarak S Alqahtani, Salmah M Alharbi, Bandar Alamri, Muayyad Alhefzi, Adel Alawwadh

**Affiliations:** 1 Department of Otorhinolaryngology-Head and Neck Surgery, Aseer Central Hospital, Abha, SAU; 2 Department of Otorhinolaryngology-Head and Neck Surgery, Abha Private Hospital, Abha, SAU; 3 Department of Surgery, Faculty of Medicine, King Khalid University, Abha, SAU; 4 Department of Pediatrics, Khamis Mushayt Maternity and Children Hospital, Khamis Mushayt, SAU; 5 Department of Pediatrics, Abha Private Hospital, Abha, SAU

**Keywords:** surgical excision, temple region, child, plexiform neurofibroma, case report

## Abstract

Plexiform neurofibroma is a rare variant of neurofibromatosis type 1. Diagnosis is challenging due to the highly variable clinical presentation. Early diagnosis is essential for appropriate treatment and prevention of complications. This report describes a sporadic solitary plexiform neurofibroma in the temporal region of a seven-year-old girl. The growth of the mass began at birth and grew steadily over five years. Subsequently, the mass began to expand rapidly. The patient underwent complete surgical resection under general anesthesia. Histopathological examination revealed a plexiform neurofibroma. In conclusion, surgical excision is the gold standard for cases with symptomatic, visible, large superficial lesions.

## Introduction

Neurofibromas are slow-growing, painless, and locally infiltrating tumors that usually manifest as localized or diffuse lesions and rarely as a plexiform variety [1]. Neurofibromatosis type 1 is a genetic disorder, and the presentation of patients is extremely variable. Nearly 5%-15% of patients with NF-1 disorder manifest with the plexiform variety [2].

There are many clinical concerns for plexiform neurofibroma including the significant cosmetic disfigurement, compression of the adjacent vital structures, and the potential for malignant transformation, which occurs in about 10% of cases [3]. Plexiform neurofibroma is a rare tumor that arises from Schwann cells in the sheaths of peripheral nerves and is a variant of neurofibromatosis type 1 (NF1) [4].

The localized form, which is the most common presentation, is familiar to radiologists. The imaging appearance has been extensively reported and documented. However, the plexiform variant, which arises from multiple nerves and their branches and may infiltrate the surrounding soft tissue and skin, has been less commonly reported. The clinical concern with plexiform neurofibroma is not only the significant cosmetic disfigurement and compression of adjacent vital structures but also its potential for malignant transformation. This occurs in approximately 10% of cases [5,6].

Diagnosis is therefore challenging. Early diagnosis is crucial for effective management of symptoms and prevention of potential complications. This report describes a successful complete resection of a plexiform neurofibroma in a seven-year-old girl in whom the tumor was sporadic and solitary with no associated manifestations.

## Case presentation

A seven-year-old girl presented with a painless progressive swelling in the right temporal region of the head. The growth of the mass started at birth when the family noticed a smooth mass resembling a birthmark. Then, at the age of eight months, it was found to be a small maculopapular patch of about 3.2 cm. The mass grew steadily over the next five years. At the age of six, the mass began to grow rapidly, reaching a maximum size of 5 x 0.5 x 1.5 cm at the age of seven.

The patient was born at term, was medically and surgically uneventful, and was up to date on immunizations. There was no history of neonatal or pediatric intensive care admission. There was no family history of similar conditions.

On clinical examination, the mass was firm, smooth, not tender, or pulsatile, and not adherent to the overlying skin or underlying structures. There was some skin discoloration or discharge, along with marked hair loss in the skin surrounding the mass. There were no congenital anomalies of the ear. There was no peripheral lymphadenopathy.

**Figure 1:**
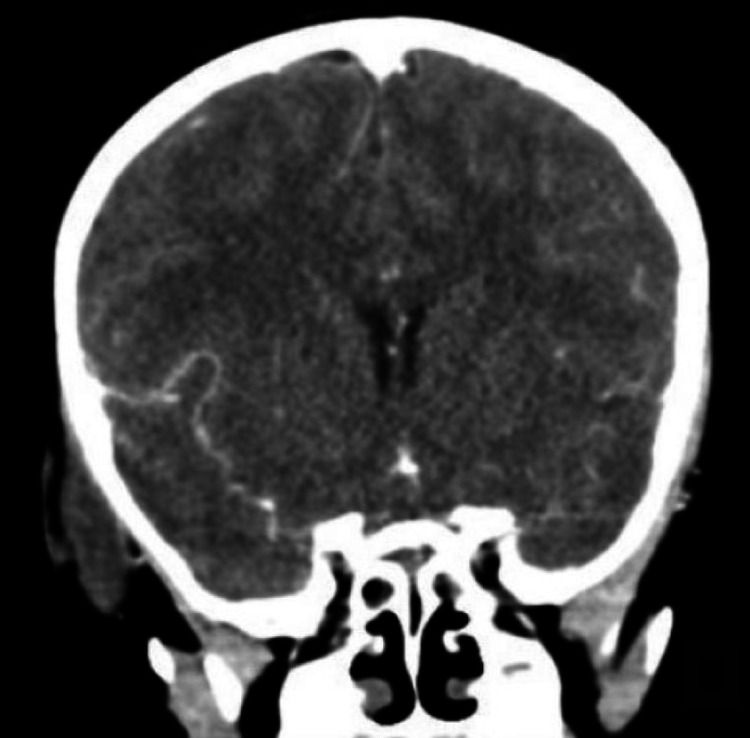
A preoperative contrast computed tomography scan showing a hypodense mass (approximately 5 cm in diameter) involving the right temple, extending to the superior parotid gland, and sparing the right external auditory canal

A preoperative contrast-enhanced computed tomography scan revealed a hypodense mass involving the right temple, extending to the superior parotid gland, and sparing the right external auditory canal. The mass was approximately 5 cm in diameter (Figure 1).

**Figure 2:**
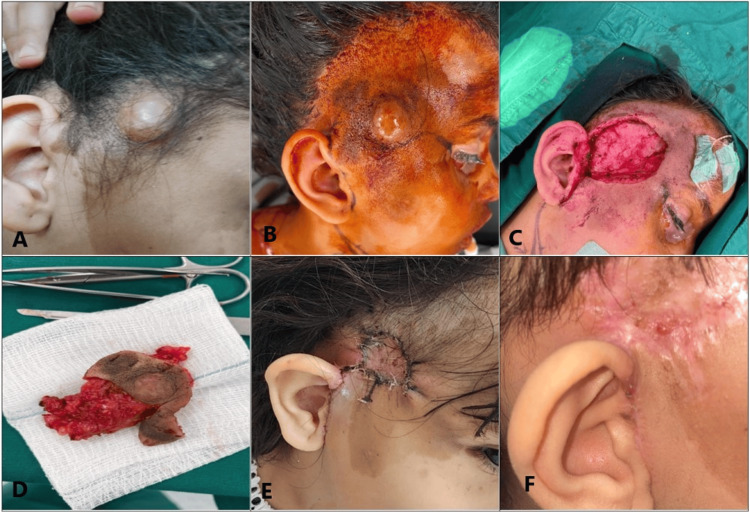
(A) Preoperative appearance of the mass. (B) Intraoperative appearance of a yellow-colored mass, having a tiny nodular surface with no skin or deeper tissue infiltration. (C) and (D) Complete excision of the mass. (E) Postoperative follow-up at one week. (F) Postoperative follow-up at two months.

The patient underwent complete surgical excision of the mass with a 1-cm safety margin under general anesthesia (Figure 2, Panel A). Intraoperatively, the patient was placed in the left lateral decubitus position (Figure 2, Panel B). An incision was made to delineate the lesion, and the subcutaneous fat was dissected (Figure 2, Panel C). The mass was yellow with a small nodular surface (Figure 2, Panel D). There was no infiltration of the subcutaneous or deeper tissues. The facial nerve was fully exposed and intact. After the complete excision of the mass, the surgical site was covered with a full-thickness skin graft. Postoperative follow-up was performed twice: one week postoperatively (Figure 2, Panel E) and two months later (Figure 2, Panel F).

**Figure 3:**
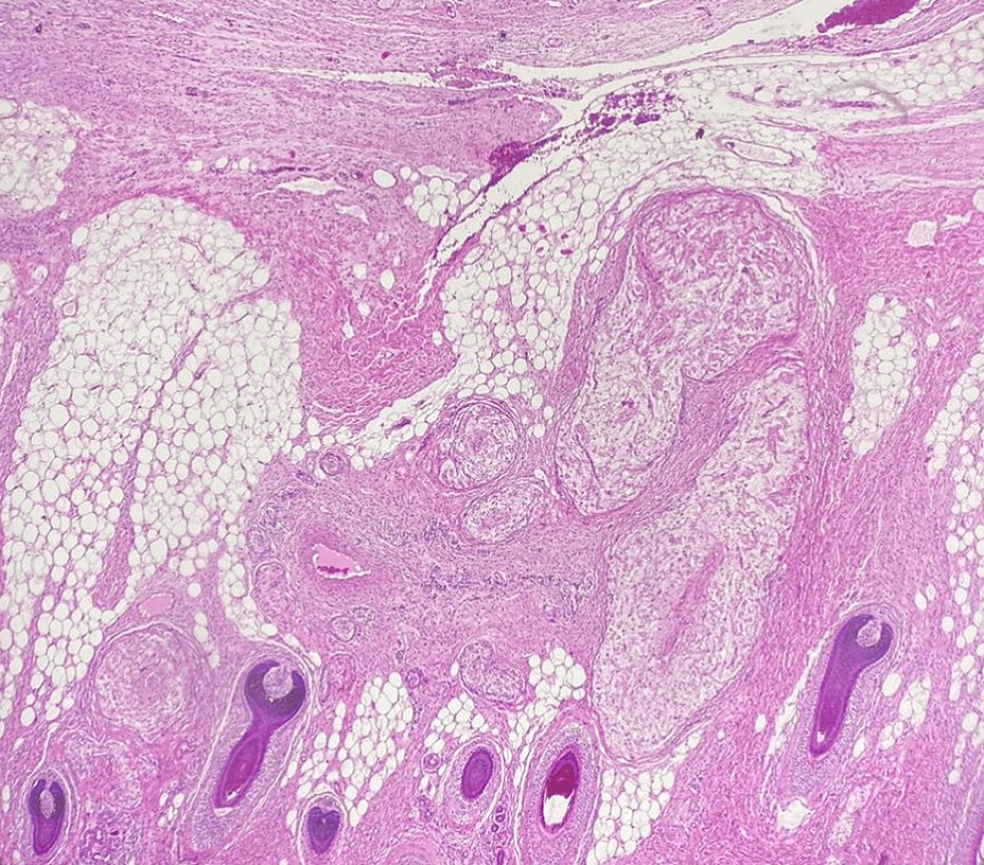
Tissue excised from the right parotid gland showing peripheral nerve sheath tumor consistent with plexiform neurofibroma Hematoxylin and eosin (H&E) staining, 40x.

The patient had a follow-up with pediatric neurology and plastic surgery, which she completed without further need for management. The family refused genetic screening due to their financial issues. A gross histopathological examination of the resected mass revealed an irregular soft gray tumor measuring 0.5 x 3.0 x 1.5 cm. Microscopic examination documented a benign peripheral nerve sheath tumor and was consistent with a plexiform neurofibroma (Figure 3).

## Discussion

Plexiform neurofibroma is a benign tumor that has the potential to develop into cancer. It is known for its rare incidence of one in 3500 births per year [7]. It is a characteristic feature of NF1, an autosomal dominant disorder caused by a mutation in the NF1 tumor suppressor gene located on chromosome 17 at locus 17q11.2 [8].

Plexiform neurofibroma is common in children and tends to progress over time. The risk of developing this tumor is significantly higher in people with a family history of NF1 [9]. However, there is a high frequency of new mutations, and almost half of NF1 cases can occur sporadically, where there is no known family history of the condition [10].

Plexiform neurofibromas may develop along one or more peripheral nerves [2]. Symptoms can vary depending on the location and size of the tumor. Disfigurement is usually the main complaint of visible plexiform neurofibromas on the skin. Compression of motor or sensory nerves by the tumor can lead to chronic pain or discomfort in the affected area, with possible functional impairment [11].

The clinical presentation of NF1 exhibits a broad spectrum with high variability between individuals and even within the same family. This makes diagnosis challenging. The diagnosis of NF1 requires the presence of two of the following features in the same individual: affected first-degree relative, at least six café-au-lait macules greater than 1.5 cm after puberty or greater than 0.5 cm before puberty, axillary or inguinal freckling, at least two neurofibromas of any type or at least one plexiform neurofibroma, optic nerve glioma, at least two iris hamartomas, and a characteristic bone lesion [12].

The diagnosis of plexiform neurofibroma involves a combination of a thorough clinical assessment, imaging studies to determine the extent of the tumor, and genetic testing [13]. Histopathological examination of the excisional biopsy helps to diagnose the type of tumor. The multinodular enlargement on gross examination is characteristic of plexiform neurofibroma. However, peripheral nerve neurofibroma may be a solitary tumor [14].

Management of plexiform neurofibromas includes conservative, surgical, and medical treatment. Surgery is the gold standard for symptomatic cases with large superficial lesions that can be completely excised. However, surgery carries some risks such as bleeding, nerve damage, and scarring [15]. In the present case, complete excision of the mass was performed for aesthetic reasons and to prevent further compression or invasion of the facial nerve or other structures. Postoperative follow-up showed complete healing with no complications.

The potential for recurrence, particularly in children, malignant transformation, and the high risk of cognitive dysfunction necessitate long-term follow-up [16,17]. The estimated lifetime risk of malignant transformation is 15.8% [18], although this risk is estimated to be as low as 0.5% in childhood [19].

## Conclusions

Plexiform neurofibroma is a benign, progressive tumor of the peripheral nerves with a potential risk of malignant transformation. Complete excision is the gold standard for cases with symptomatic, visible, large superficial lesions.
